# Supplementary material for: Validity of Clinical Symptoms Score to Discriminate Patients with COVID-19 from Common Cold Out-Patients in General Practitioner Clinics in Japan
Source: J Clin Med. 2021 Feb 19;10(4):854. doi: 10.3390/jcm10040854 (PMC7922190; doi:10.3390/jcm10040854)
Supplement: Supplementary file 1 [file jcm-10-00854-s001.pdf]

Table S1. STROBE Statement—Checklist of items that should be included in reports of ***case-control studies.***

	Item No	Recommendation
**Title and abstract**	1	(*a*) Indicate the study's design with a commonly used term in the title or the abstract **(p. 1)**
		(*b*) Provide in the abstract an informative and balanced summary of what was done and what was found **(p. 1)**
		**Introduction**
Back-ground/rationale	2	Explain the scientific background and rationale for the investigation being reported **(pp. 1-2 "Intro-duction 1st and 2nd paragraph")**
Objectives	3	State specific objectives, including any prespecified hypotheses **(p. 2 "Introduction 3rd and 4th para-graph")**
		**Methods**
Study design	4	Present key elements of study design early in the paper **(p. 2 "2.1. Study design and setting")**
Setting	5	Describe the setting, locations, and relevant dates, including periods of recruitment, exposure, follow-up, and data collection **(p. 2 "2.1. Study design and setting")**
Participants	6	(*a*) Give the eligibility criteria, and the sources and methods of case ascertainment and control selec-tion. Give the rationale for the choice of cases and controls **(p. 2 "2.1. Study design and setting")**
		(*b*) For matched studies, give matching criteria and the number of controls per case **(N/A)**
Variables	7	Clearly define all outcomes, exposures, predictors, potential confounders, and effect modifiers. Give diagnostic criteria, if applicable **(p. 2 "2.2. Possible factors to discriminate COVID-19")**
Data sources/ meas-urement	8*	For each variable of interest, give sources of data and details of methods of assessment (measurement). Describe comparability of assessment methods if there is more than one group **(p. 3 "2.3. Statistical analysis")**
Bias	9	Describe any efforts to address potential sources of bias **(N/A)**
Study size	10	Explain how the study size was arrived at **(p. 2 "2.1. Study design and setting")**
Quantitative varia-bles	11	Explain how quantitative variables were handled in the analyses. If applicable, describe which group-ings were chosen and why **(p. 3 "2.3. Statistical analysis")**
Statistical methods	12	(*a*) Describe all statistical methods, including those used to control for confounding **(p. 3 "2.3. Statis-tical analysis")**
		(*b*) Describe any methods used to examine subgroups and interactions **(N/A)**
		(*c*) Explain how missing data were addressed **(N/A)**
		(*d*) If applicable, explain how matching of cases and controls was addressed **(p. 3 "2.3. Statistical analysis")**
		(*e*) Describe any sensitivity analyses **(p. 3 "2.3. Statistical analysis")**
		**Results**
Participants	13*	(a) Report numbers of individuals at each stage of study—eg numbers potentially eligible, examined for eligibility, confirmed eligible, included in the study, completing follow-up, and analysed **(pp. 3-4 "3.1. Characteristics of the study" +table 1)**
		(b) Give reasons for non-participation at each stage **(N/A)**
		(c) Consider use of a flow diagram **(N/A)**
Descriptive data	14*	(a) Give characteristics of study participants (eg demographic, clinical, social) and information on ex-posures and potential confounders **(pp. 3-4 "3.1. Characteristics of the study" +table 1, 2)**
		(b) Indicate number of participants with missing data for each variable of interest **(N/A)**
Outcome data	15*	Report numbers in each exposure category, or summary measures of exposure **(pp. 3-4 "3.1. Charac-teristics of the study" + table 1,2)**
Main results	16	(*a*) Give unadjusted estimates and, if applicable, confounder-adjusted estimates and their precision (eg, 95% confidence interval). Make clear which confounders were adjusted for and why they were includ-ed **(pp. 5-7 "3.2. Association between possible factors and COVID-19" + table 3)**
		(*b*) Report category boundaries when continuous variables were categorized **(pp. 3-4 "3.1. Character-istics of the study" + table 2)**
		(*c*) If relevant, consider translating estimates of relative risk into absolute risk for a meaningful time period **(N/A)**
Other analyses	17	Report other analyses done—eg analyses of subgroups and interactions, and sensitivity analyses **(p. 7 "3.3. Development of the COVID-19 scoring system" + table 4, 5)**
		**Discussion**
Key results	18	Summarise key results with reference to study objectives **(p. 9 "Discussion 1st paragraph")**
Limitations	19	Discuss limitations of the study, taking into account sources of potential bias or imprecision. Discuss both direction and magnitude of any potential bias **(pp. 9-10 "Discussion 4th paragraph")**
Interpretation	20	Give a cautious overall interpretation of results considering objectives, limitations, multiplicity of anal-

		yses, results from similar studies, and other relevant evidence **(p. 9 "Discussion 2nd, 3rd paragraph")**
Generalisability	21	Discuss the generalisability (external validity) of the study results **(p. 10 "Discussion 5th paragraph")**
		**Other information**
Funding	22	Give the source of funding and the role of the funders for the present study and, if applicable, for the original study on which the present article is based **(p. 10)**
